# Consistency of the determinants of early initiation of breastfeeding in Ghana: insights from four Demographic and Health Survey datasets

**DOI:** 10.1093/inthealth/ihaa017

**Published:** 2020-04-17

**Authors:** Precious A Duodu, Henry O Duah, Veronica M Dzomeku, Adwoa B Boamah Mensah, Josephine Aboagye Mensah, Ernest Darkwah, Pascal Agbadi

**Affiliations:** Department of Nursing, Faculty of Allied Health Sciences, College of Health Sciences, Kwame Nkrumah University of Science and Technology, Private Mail Bag, Kumasi, Ghana; Research Department, Foundation of Orthopaedic and Complex Spine Hospital,Post Office Box KD 779 Kanda, Accra, Ghana; Department of Nursing, Faculty of Allied Health Sciences, College of Health Sciences, Kwame Nkrumah University of Science and Technology, Private Mail Bag, Kumasi, Ghana; Department of Nursing, Faculty of Allied Health Sciences, College of Health Sciences, Kwame Nkrumah University of Science and Technology, Private Mail Bag, Kumasi, Ghana; Child Health Directorate, Komfo Anokye Teaching Hospital, P.O.Box 1934 Adum-Kumasi, Ghana; Department of Psychology, University of Ghana, Post Office Box LG 84, Legon, Accra, Ghana; Department of Nursing, Faculty of Allied Health Sciences, College of Health Sciences, Kwame Nkrumah University of Science and Technology, Private Mail Bag, Kumasi, Ghana

**Keywords:** early initiation of breastfeeding, Demographic and Health Survey data, sub-Saharan Africa, Ghana

## Abstract

**Background:**

Early initiation of breastfeeding (EIBF) is a key strategy in averting neonatal deaths. However, studies on the facilitators and risk factors for EIBF are rare in Ghana. We examined trends in EIBF and its major facilitators and risk factors in Ghana using data from Demographic and Health Surveys from 1998 to 2014.

**Methods:**

We used complete weighted data of 3194, 3639, 2909 and 5695 pairs of mothers ages 15–49 y and their children ages 0–5 y in the 1998, 2003, 2008 and 2014 surveys, respectively. We accounted for the complex sampling used in the surveys for both descriptive statistics and multiple variable risk ratio analysis.

**Results:**

The proportion of children who achieved EIBF increased by about 2.5 times from 1998 to 2003 and there was a marginal increase in the proportion of children who achieved EIBF between 2003 and 2014. Children born by caesarean section were at higher risk of being breastfed later than 1 h across all four surveys. Being born in the Upper East Region (compared with the Western Region) of Ghana facilitated EIBF in 2003 and 2008.

**Conclusions:**

The study revealed that the current estimate of the proportion of children achieving EIBF in Ghana was 55.1%, and delivery by caesarean section and region of residence consistently predicted the practice of EIBF in Ghana.

## Introduction

Evidence suggests that delayed initiation of breastfeeding beyond 1 h after birth is associated with higher risks of neonatal mortality and morbidity.^1–3^ This is a major bottleneck to the achievement of Sustainable Development Goal 3.2,^4^ which makes the promotion of early initiation of breastfeeding (EIBF), putting a child to the mother’s breast within the first hour of birth, a key public health child survival strategy.^5^

EIBF benefits both the child and mother in several ways. EIBF is a key predictor of exclusive breastfeeding and longer duration of breastfeeding.^6^ EIBF activates early milk let-down and ensures that the baby receives the colostrum, which is rich in nutrients and antibodies to prevent infections in infants, meriting the name baby’s ‘first vaccine’.^7^ EIBF, through skin-to-skin contact, reduces the experience of birth stress for the child and the mother, and controls the child’s temperature, thus preventing hypothermia and hypoglycaemia, which are threats to the survival of the neonate in the first week of life.^8^ EIBF facilitates strong mother–child bonding and significantly contributes to the child’s cognitive development.^9^ For the mother, EIBF increases uterine activity, leading to possible risk reductions of heavy bleeding and infections and helps to expel the placenta.^10^

Despite the enormous benefits of EIBF to maternal and child health and well-being, not all children are breastfed within the first hour of birth. Global estimates for the year 2017 indicated that only two of five children were breastfed early.^7^ Estimates of the proportion of children who were breastfed early in West and Central Africa was 40% and that of Eastern and Southern Africa was 65%.^7^ However, Burundi has a positive record of nearly 9 in 10 children being breastfed within the first hour of birth.^7^ A 2017 multiple indicator cluster survey report stated that about 52% of children in Ghana were breastfed early, suggesting that about 5 of every 10 children in Ghana were denied the benefits of EIBF.^11^

Although Ghana has made significant progress on multiple maternal and child health outcomes,^12^ it is clear that the country was unable to achieve the Millennium Development Goals (MDG) and there are doubts concerning Ghana’s ability to achieve the new Sustainable Development Goals (SDGs) by 2030.^12^ By the end of 2015, Ghana was expected to achieve maternal mortality and under-5 mortality rates of 190 deaths per 100 000 live births and 40 per 1000 live births, respectively. The records reveal that Ghana significantly reduced maternal mortality from 760 to 319 per 100 000 live births and under-5 mortality from 108 to 60 per 1000 live births between 1990 and 2015.^12^ Given Ghana’s historical records regarding the achievement of maternal- and child health-related goals, it is worth investigating when Ghana is likely to meet its ambitious target of ensuring that about 85% of its newborns are breastfed within the first hour of birth.^13^

Studies have revealed that multiple sociocultural, economic and demographic factors promote or prevent EIBF.^14–17^ Some of these factors include the gender of the baby, birth order, place of birth, type of delivery and birth, maternal age, maternal education and religious affiliation.^14–17^ Certain household factors, including the wealth index, rural–urban locality and region of residence, have also been cited as possible determinants of EIBF.^14^^,^^15^^,^^18^ Almost all these studies were done outside Ghana, indicating that current studies on sociodemographic and economic predictors of EIBF in Ghana are rare. An extant and decade-old qualitative study suggested that delivery at a health facility promotes EIBF, while perceptions such as insufficient breast milk and the need for mother and child to rest after delivery and the performing of post-birth activities such as bathing were identified as barriers to EIBF.^19^

To date, no study has investigated the trend in EIBF among children and forecasted when Ghana may achieve its own EIBF target using nationally representative data. Therefore we conducted this study using nationally representative Demographic and Health Survey (DHS) data from 1998 to 2014 to present the trends in the proportion of EIBF and determine Ghana’s progress in achieving its EIBF target as well as examine the major facilitators and risk factors associated with EIBF in Ghana. We believe that this approach helps provide more comprehensive and representative insights into the state of EIBF in the country.

## Methods

### Study population and sample

In this study we analysed survey data on children from the Ghana Demographic and Health Survey (GDHS) at four time points (1998, 2003, 2008 and 2014). The GDHS is conducted every 5 y in Ghana, starting from 1988. We used datasets of the years mentioned because they had questions on EIBF. The GDHS employed a stratified two-stage sampling design consisting of the choosing of enumeration areas as clusters. Clusters are randomly selected from both rural and urban areas. Households were then randomly selected from each selected cluster. The total number of clusters and households varied across the survey rounds. In each selected household, data were collected on 3298, 3844, 2992 and 5884 pairs of mothers ages 15–49 y and their children 0–5 y in 1998, 2003, 2008 and 2014, respectively. After adjusting for the complex samples design and sampling weight, the study samples became 3194, 3639, 2909 and 5695 cases for the 1998, 2003, 2008 and 2014 survey rounds, respectively. Thirty-four (1.06%), 62 (1.70%), 61 (2.10%) and 23 (0.40%) children with missing information on multiple variables were removed from the 1998, 2003, 2008 and 2014 datasets, respectively, during the multiple variable risk ratio modelling. Therefore, there were complete records on weighted samples of 3160, 3639, 2848 and 5672 pairs of mothers and their children in the 1998, 2003, 2008 and 2014 datasets, respectively.

### Outcome variables

The main outcome of interest was the EIBF. This is defined as breastfeeding within the first hour of birth. As part of the survey, mothers were asked whether they breastfed their children at all. Subsequently those who breastfed were asked if breastfeeding was done within the first hour of birth. In our study, children who were breastfed within the first hour of birth were value labelled as 1 and those who breastfed later than an hour or did not breastfeed at all were value labelled as 0.

### Explanatory variables

Explanatory variables include the gender of the baby, type of birth, childbirth order, birth size, delivery by caesarean section, assisted birth deliveries, maternal age, education, media exposure (reading newspaper, watching television, listening to the radio), place and region of residence and household wealth index. We focused on these variables as possible predictors due to evidence in the literature that suggests that these factors have a predictive relationship with EIBF.^14–17^ The idea was to test these variables and see how they apply in the Ghanaian context when nationally representative data are used. Except for the religion, childbirth order, type of birth, place of delivery and assisted deliveries variables, all the other variables were used as operationalized in the DHS datasets. We recoded the religion variable as follows: no religion and other = no religion + other; Catholic, Anglican, Methodist, Presbyterian, Pentecostal/charismatic and other Christian = Christian; Islam = Islam; and Traditional and Spiritualist = Traditional/spiritualist. The values of the childbirth order variable in the dataset were reported as discrete values and were recoded as follows: 1 = first, 2 = second, 3 = third, 4 = fourth and 5+ = fifth or more. The type of birth was recoded as follows: single birth = singleton; first of multiple, second of multiple, third of multiple, fourth of multiple or fifth of multiple = multiple births. The place of delivery variable was recoded based on the DHS’s predetermined categorization as follows: respondent’s home and other home = home; government hospital, government health centre/clinic, government health post/Community-based Health Planning and Services = public; private hospital, clinic, family planning/Planned Parenthood Association of Ghana clinic, mobile clinic, maternity home and other private medical = private.

In 1998, six variables in the dataset measured assisted deliveries with yes = 1 or no = 0 response: assisted deliveries included by a (A) doctor, (B) nurse, (C) midwife, (D) trained traditional birth attendant, (E) traditional birth attendant (TBA) or (F) others. The COMPUTE function in the Statistical Package for Social Sciences (SPSS) software (version 21; IBM, Armonk, NY, USA) was used to create a new variable, assisted deliveries: Compute AssistedDeliveries = 0. If (A = 1) AssistedDeliveries = 1. If (B = 1) | (C = 1) AssistedDeliveries = 2. If (D = 1) | (E = 1) AssistedDeliveries = 3. If (F = 1) AssistedDeliveries = 4. EXECUTE. The numerical categories stand for the following: no assistance, 0; doctor, 1; midwife/nurse, 2; TBA, 3; others, 4. We followed the same variable computing method to create the ‘assisted deliveries’ variable for the 2003, 2008 and 2014 survey datasets.

### Statistical analysis

Descriptive statistics and multivariate risk ratio estimates for the selected predictors of EIBF were performed using SPSS version 21 and Stata version 14 (StataCorp, College Station, TX, USA), respectively. The GDHS is a multistage stratified design survey, so we adopted a complex sampling design analysis. By adopting this method of analysis, we eliminated the possibility of the underestimation of the standard errors (SEs) associated with the confidence intervals of the regression coefficients when complex sample designs are disregarded in the analysis. Before performing the descriptive statistics in SPSS, we first created a complex sample plan file using the sample weight, primary sampling unit and sample strata variables. In Stata, we adjusted for the complex sample nature of the datasets by using the svyset command before performing the multivariate risk ratio. We estimated the adjusted risk ratios using the generalized linear model, setting the family to ‘Poisson’ and the link to ‘log’. Risk ratios appropriately estimate the risk in situations where the outcome of interest is not rare, and it has been recommended over the standard logistic regression model that reports odds ratios, which could bias the risk estimate in a similar situation.^20–22^

**Table 1:** Complex sample descriptive statistics estimates of study variables

Variables	1998	2003	2008	2014
**Breastfed early**				
Yes	574 (18.0)	1627 (44.7)	1493 (51.3)	3138 (55.1)
No	2620 (82.0)	2012 (55.3)	1416 (48.7)	2557 (44.9)
*Child factors*				
**Gender of the baby**				
Boy	1573 (49.2)	1841 (50.6)	1510 (51.9)	2970 (52.2)
Girl	1621 (50.8)	1798 (49.4)	1399 (48.1)	2725 (47.8)
**Type of birth**				
Singleton	3052 (95.6)	3494 (96.0)	2783 (95.7)	5403 (94.9)
Multiple birth	142 (4.4)	145 (4.0)	126 (4.3)	292 (5.1)
**Birth order**				
1	749 (23.5)	820 (22.5)	688 (23.7)	1369 (24.0)
2	651 (20.4)	707 (19.4)	613 (21.1)	1194 (21.0)
3	471 (14.7)	564 (15.5)	494 (17.0)	987 (17.3)
4	380 (11.9)	452 (12.4)	395 (13.6)	786 (13.8)
5+	943 (29.5)	1097 (30.1)	719 (24.7)	1358 (23.9)
**Birth size**				
Very large	345 (10.9)	438 (12.2)	644 (22.4)	1013 (17.8)
Larger than average	1499 (47.3)	1060 (29.5)	932 (32.5)	1888 (33.3)
Average	917 (28.9)	1434 (39.9)	898 (31.3)	1881 (33.1)
Smaller than average	361 (11.4)	419 (11.7)	273 (9.5)	640 (11.3)
Very small	49 (1.5)	241 (6.7)	120 (4.2)	256 (4.5)
Missing cases	23	46	42	18
**Caesarean section**				
Yes	127 (4.0)	134 (3.7)	201 (6.9)	729 (12.8)
No	3047 (96.0)	3476 (96.3)	2706 (93.0)	4966 (87.2)
Missing cases	20	29	3	
**Place of delivery**				
Home	1778 (55.9)	1942 (53.7)	1223 (42.2)	1514 (26.6)
Public hospital	1045 (32.9)	1320 (36.5)	1408 (48.6)	3700 (65.1)
Private hospital	358 (11.2)	354 (9.8)	267 (9.2)	480 (8.4)
Missing cases	14	23	12	1
**Delivery assistance**				
No assistance	163 (5.1)	103 (2.8)	88 (3.0)	165 (2.9)
Doctor	98 (3.1)	115 (3.2)	157 (5.4)	398 (7.0)
Midwife/nurse	1300 (40.7)	1565 (43.0)	1547 (53.2)	3799 (66.7)
TBA	1339 (41.9)	1015 (27.9)	875 (30.1)	914 (16.1)
Others	295 (9.2)	842 (23.1)	242 (8.3)	419 (7.4)
*Maternal factors*				
**Age (years)**				
15–19	114 (3.6)	125 (3.4)	116 (4.0)	204 (3.6)
20–24	709 (22.2)	680 (18.7)	568 (19.5)	971 (17.0)
25–29	866 (27.1)	953 (26.2)	811 (27.9)	1421 (25.0)
30–34	615 (19.2)	810 (22.3)	593 (20.4)	1418 (24.9)
35–39	506 (15.8)	627 (17.2)	501 (17.2)	1052 (18.5)
40–44	291 (9.1)	302 (8.3)	225 (7.7)	488 (8.6)
45–49	95 (3.0)	143 (3.9)	95 (3.3)	141 (2.5)
**Education**				
None	1228 (38.5)	1466 (40.3)	952 (32.7)	1561 (27.4)
Primary	649 (20.3)	843 (23.2)	722 (24.8)	1141 (20.0)
Secondary	1287 (40.3)	1289 (35.4)	1165 (40.0)	2739 (48.1)
Post-secondary	30 (0.9)	40 (1.1)	70 (2.4)	254 (4.5)
**Read newspaper**				
Yes	318 (10.0)	328 (9.0)	315 (10.8)	654 (11.5)
No	2875 (90.0)	3307 (91.0)	2589 (89.2)	5036 (88.5)
Missing	1	4	5	5
**Listen to radio**				
Yes	1699 (53.2)	3044 (83.7)	2315 (79.7)	4655 (81.7)
No	1495 (46.8)	594 (16.3)	591 (20.3)	1040 (18.3)
Missing	1	1	3	
**Watch television**				
Yes	1228 (38.5)	1686 (46.3)	1490 (51.4)	1712 (30.1)
No	1964 (61.5)	1951 (53.7)	1411 (48.6)	3983 (69.9)
Missing	3	2	8	
**Religion**				
None + other	301 (9.4)	238 (6.5)	130 (4.5)	239 (4.2)
Christian	2245 (70.3)	2593 (71.3)	2034 (70.0)	4303 (75.6)
Muslim	368 (11.5)	655 (18.0)	552 (19.0)	970 (17.0)
Traditionalist	280 (8.8)	151 (4.1)	191 (6.6)	183 (3.2)
Missing		3	2	
*Residential factors*				
**Place of residence**				
Urban	774 (24.2)	1204 (33.1)	1104 (37.9)	2563 (45.0)
Rural	2421 (75.8)	2435 (66.9)	1806 (62.1)	3132 (55.0)
**Wealth index**				
Poorest		941 (25.9)	744 (25.6)	1263 (22.2)
Poorer		809 (22.2)	641 (22.0)	1196 (21.0)
Middle		721 (19.8)	549 (18.9)	1114 (19.6)
Richer		617 (16.9)	560 (19.3)	1074 (18.9)
Richest		551 (15.1)	415 (14.3)	1048 (18.4)
**Region**				
Western	413 (12.9)	367 (10.1)	271 (9.3)	574 (10.1)
Central	379 (11.9)	304 (8.4)	292 (10.1)	622 (10.9)
Greater Accra	329 (10.3)	390 (10.7)	346 (11.9)	880 (15.5)
Volta	338 (10.6)	298 (8.2)	244 (8.4)	436 (7.7)
Eastern	430 (13.5)	362 (9.9)	254 (8.7)	532 (9.3)
Ashanti	514 (16.1)	685 (18.8)	545 (18.7)	1065 (18.7)
Brong Ahafo	260 (8.1)	401 (11.0)	272 (9.3)	497 (8.7)
Northern	232 (7.3)	500 (13.7)	456 (15.7)	709 (12.5)
Upper West	100 (3.1)	118 (3.2)	82 (2.8)	152 (2.7)
Upper East	199 (6.2)	215 (5.9)	148 (5.1)	227 (4.0)

Values are presented as n (%).

**Table 2:** Adjusted risk ratio model results from complex sample regression analysis

Variables	1998	2003	2008	2014
**Gender of the baby**				
Boy	1.00 (reference)	1.00 (reference)	1.00 (reference)	1.00 (reference)
Girl	1.10 (0.95–1.27)	1.01 (0.94–1.08)	1.00 (0.93–1.08)	1.03 (0.98–1.09)
**Type of birth**				
Singleton	1.00 (reference)	1.00 (reference)	1.00 (reference)	1.00 (reference)
Multiple birth	0.35* (0.19–0.62)	0.71 (0.49–1.03)	0.83 (0.58–1.18)	1.05 (0.89–1.23)
**Birth order**				
1	1.00 (reference)	1.00 (reference)	1.00 (reference)	1.00 (reference)
2	1.34* (1.04–1.74)	1.03 (0.94–1.13)	1.02 (0.93–1.12)	1.11* (1.03–1.20)
3	1.95* (1.47–2.59)	1.05 (0.92–1.20)	1.01 (0.89–1.15)	1.12* (1.01–1.25)
4	2.10* (1.54–2.85)	1.11 (0.95–1.29)	0.95 (0.80–1.12)	1.18* (1.04–1.34)
5+	1.99* (1.44–2.76)	1.02 (0.86–1.20)	0.93 (0.78–1.11)	1.19* (1.04–1.37)
**Birth size**				
Very large	1.00 (reference)	1.00 (reference)	1.00 (reference)	1.00 (reference)
Larger than average	0.79* (0.63–0.98)	1.10 (0.97–1.24)	0.98 (0.88–1.11)	1.08 (0.97–1.21)
Average	0.84 (0.66–1.07)	1.05 (0.93–1.19)	1.01 (0.89–1.14)	1.08 (0.97–1.21)
Smaller than average	0.80 (0.59–1.09)	1.02 (0.87–1.20)	0.87 (0.73–1.03)	0.96 (0.84–1.11)
Very small	0.90 (0.50–1.62)	0.94 (0.78–1.14)	1.05 (0.85–1.29)	1.01 (0.84–1.21)
**Caesarean section**				
No	1.00 (reference)	1.00 (reference)	1.00 (reference)	1.00 (reference)
Yes	0.42* (0.25–0.71)	0.48* (0.33–0.71)	0.41* (0.28–0.60)	0.53* (0.43–0.65)
**Place of delivery**				
Home	1.00 (reference)	1.00 (reference)	1.00 (reference)	1.00 (reference)
Public hospital	1.18 (0.74–1.87)	1.29 (1.00–1.68)	0.89 (0.70–1.13)	0.90 (0.72–1.12)
Private hospital	1.14 (0.70–1.83)	1.11 (0.85–1.45)	0.75* (0.57–0.98)	0.96 (0.75–1.22)
**Delivery assistance**				
No assistance	1.00 (reference)	1.00 (reference)	1.00 (reference)	1.00 (reference)
Doctor	2.24* (1.08–4.67)	1.49 (0.86–2.59)	1.58 (0.87–2.87)	1.52* (1.05–2.19)
Nurse/midwife	2.04* (1.05–3.94)	1.47 (0.91–2.37)	1.88* (1.14–3.11)	1.63* (1.20–2.22)
TBA	1.45 (0.86–2.45)	1.44 (0.94–2.19)	1.38 (0.90–2.13)	1.36* (1.06–1.74)
Other	1.06 (0.58–1.94)	1.35 (0.89–2.04)	1.35 (0.85–2.15)	0.94 (0.69–1.28)
**Age (years)**				
15–19	1.00 (reference)	1.00 (reference)	1.00 (reference)	1.00 (reference)
20–24	0.79 (0.50–1.26)	1.17 (0.89–1.53)	1.07 (0.82–1.40)	0.84* (0.73–0.97)
25–29	0.77 (0.48–1.24)	1.26 (0.97–1.63)	1.06 (0.82–1.37)	0.84* (0.72–0.97)
30–34	0.57* (0.34–0.95)	1.21 (0.92–1.59)	1.08 (0.82–1.42)	0.82* (0.69–0.97)
35–39	0.55 (0.32–0.94)	1.26 (0.93–1.70)	1.05 (0.79–1.39)	0.74* (0.60–0.90)
40–44	0.52 (0.29–0.96)	1.47 (1.08–2.00)	1.03 (0.72–1.46)	0.73* (0.58–0.92)
45–49	0.42 (0.20–0.90)	1.17 (0.81–1.68)	0.77 (0.50–1.20)	0.79 (0.61–1.04)
**Education**				
No formal education	1.00 (reference)	1.00 (reference)	1.00 (reference)	1.00 (reference)
Primary	0.95 (0.75–1.20)	1.06 (0.91–1.23)	0.96 (0.83–1.11)	0.93 (0.83–1.05)
Secondary	0.97 (0.78–1.22)	1.10 (0.96–1.27)	0.93 (0.78–1.09)	1.02 (0.92–1.12)
Post-secondary	1.28 (0.66–2.50)	1.46 (0.97–2.21)	1.00 (0.70–1.43)	1.17 (0.95–1.43)
**Read newspaper**				
No	1.00 (reference)	1.00 (reference)	1.00 (reference)	1.00 (reference)
Yes	0.97 (0.72–1.30)	0.85 (0.71–1.03)	0.93 (0.78–1.12)	0.97 (0.86–1.09)
**Listen to radio**				
No	1.00 (reference)	1.00 (reference)	1.00 (reference)	1.00 (reference)
Yes	1.00 (0.84–1.18)	1.07 (0.94–1.23)	0.98 (0.86–1.13)	0.99 (0.90–1.09)
**Watch television**				
No	1.00 (reference)	1.00 (reference)	1.00 (reference)	1.00 (reference)
Yes	1.04 (0.86–1.26)	1.10 (0.99–1.23)	0.93 (0.82–1.05)	0.94 (0.84–1.05)
**Religion**				
None/others	1.00 (reference)	1.00 (reference)	1.00 (reference)	1.00 (reference)
Christian	0.95 (0.70–1.29)	0.92 (0.77–1.10)	1.17 (0.92–1.50)	0.96 (0.81–1.14)
Muslim	0.93 (0.65–1.33)	0.92 (0.76–1.12)	1.16 (0.88–1.52)	0.91 (0.75–1.11)
Traditionalist/spiritualist	0.85 (0.55–1.32)	0.80 (0.58–1.09)	0.75 (0.54–1.04)	0.84 (0.60–1.18)
**Wealth index**				
Poorest	1.00 (reference)	1.00 (reference)	1.00 (reference)	1.00 (reference)
Poorer	-	1.18* (1.02–1.36)	0.85 (0.73–1.00)	1.02 (0.91–1.14)
Middle	-	1.06 (0.89–1.26)	0.97 (0.80–1.18)	1.03 (0.88–1.20)
Richer	-	1.04 (0.82–1.30)	1.05 (0.85–1.28)	1.17 (0.99–1.38)
Richest	-	1.09 (0.83–1.43)	1.12 (0.87–1.44)	1.15 (0.93–1.41)
**Place of residence**				
Urban	1.00 (reference)	1.00 (reference)	1.00 (reference)	1.00 (reference)
Rural	0.71* (0.56–0.91)	1.07 (0.88–1.29)	0.95 (0.83–1.09)	0.99 (0.88–1.11)
**Region**				
Western	1.00 (reference)	1.00 (reference)	1.00 (reference)	1.00 (reference)
Central	0.95 (0.65–1.38)	0.36* (0.22–0.61)	1.07 (0.84–1.36)	1.01 (0.87–1.17)
Greater Accra	1.42 (0.98–2.05)	1.26 (0.90–1.75)	0.87 (0.70–1.07)	0.82* (0.67–0.99)
Volta	0.99 (0.69–1.43)	1.34 (0.92–1.95)	1.01 (0.80–1.29)	0.78* (0.63–0.96)
Eastern	0.91 (0.59–1.39)	1.12 (0.78–1.59)	0.86 (0.67–1.08)	0.85 (0.71–1.01)
Ashanti	1.27 (0.91–1.77)	1.47* (1.07–2.01)	0.90 (0.73–1.12)	0.79* (0.65–0.96)
Brong Ahafo	0.59 (0.35–1.01)	1.46* (1.05–2.02)	0.79* (0.63–0.99)	1.00 (0.84–1.19)
Northern	1.27 (0.82–1.96)	1.87* (1.34–2.60)	0.92 (0.71–1.19)	1.12 (0.92–1.36)
Upper West	1.01 (0.67–1.53)	0.65 (0.40–1.06)	1.12 (0.86–1.45)	0.69* (0.56–0.86)
Upper East	0.43* (0.23–0.84)	2.97* (2.15–4.12)	1.32* (1.03–1.67)	1.17 (0.97–1.40)
**Intercept**	0.15 (0.07–0.33)	0.13 (0.07–0.24)	0.38 (0.20–0.70)	0.50 (0.34–0.73)
**Model details**				
Number of strata	20	20	20	20
Number of PSUs	397	409	408	427
Number of observations	3259	3783	2932	5862
Population size	31 606 507	35 775 287	28 483 848	56 720 818
Design df	377	389	388	407

Values are presented as the adjusted risk ratio (95% confidence interval).

PSU: primary sampling unit.

*Risk ratio estimates are significant at 95% CI.

**Figure 1:**
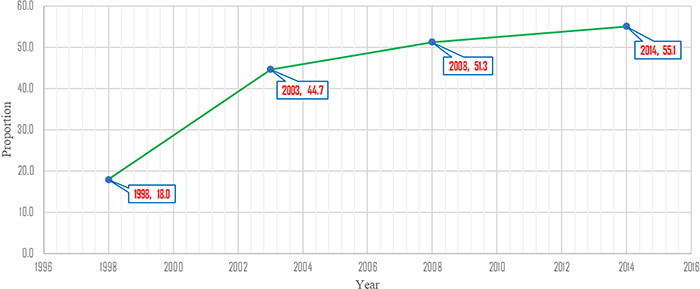
Trends in EIBF in Ghana from 1998 to 2014

## Results

### Trends in early breastfeeding in Ghana from 1998 to 2014

Generally, there was an increase in the proportion of EIBF in Ghana from 1998 to 2014 (Figure 1). The highest proportion of EIBF was recorded in 2014. The proportion of children who achieved EIBF increased by about 2.5 times from 1998 to 2003 (Figure 1). We observed that there was a marginal increase in the proportion of children who achieved EIBF from 2003 to 2014 (Figure 1). Using the prevalence estimates for 2003, 2008 and 2014, we performed a basic forecast analysis in Excel (Microsoft, Redmond, WA, USA) to determine how long it will take Ghana to achieve its EIBF target of 85%. We excluded the 1998 EIBF prevalence from the forecast analysis because it is extremely far away from the prevalence of the other years. The forecast analysis revealed that, should the trend remain the same, Ghana may achieve its EIBF target in 2044. The DHS programme is expected to collect data every 5 y, so the projections were estimated based on a 5 y interval between each survey round.

**Figure 2:**
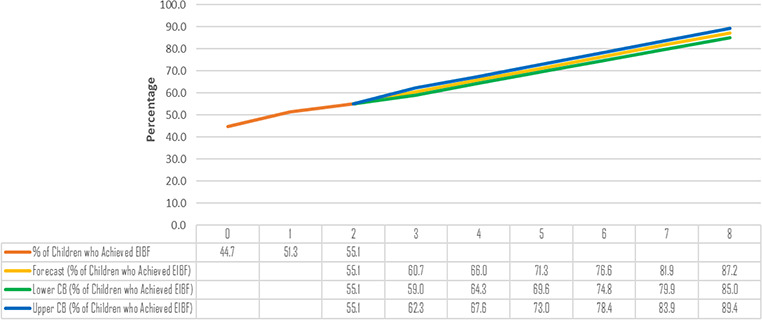
A visual forecast of when Ghana is likely to achieve its EIBF target of 85%

In Figure 2, the numbers from 0 to 8 on the horizontal axis represent each survey round: 0, 2003; 1, 2008; 2, 2014; 3, 2019; 4, 2024; 5, 2029; 6, 2034: 7, 2039; and 8, 2044. This forecast is statistically significant at the 0.10 α level. ‘CB’ in Figure 2 means confidence bound.

### Sample characteristics

Except for 1998, male children slightly outnumbered females across the survey rounds. Home delivery declined from 55.9% (1778/3194) in 1998 to 26.6% (1514/5695) in 2014. Non-assisted childbirths decreased from 5.1% (163/3194) in 1998 to 2.9% (165/5695) in 2014. The proportion of children whose mothers completed secondary school increased from 40.3% (1287/3194) in 1998 to 48.1% (2739/5695) in 2014. In the datasets, there were more children born in rural communities than in urban communities. Descriptive statistics of all study variables are presented in Table 1.

### Facilitators and risk factors of EIBF

Table 2 presents the results of the multiple variable risk ratio model for EIBF for the 1998, 2003, 2008 and 2014 surveys. The main results were that delivery by caesarean section and the region of residence were consistently associated with EIBF. Children born by caesarean section were at higher risk of being breastfed later than 1 h across all the four surveys. Except for 1998, where they were at risk, children born in the Upper East Region (compared with the Western Region) had a higher likelihood of achieving EIBF in 2003 and 2008.

Compared with non-assisted deliveries (those not assisted by health care professionals or TBAs or other types of birth attendants), childbirths assisted by a midwife or nurse facilitated EIBF more often in 1998, 2008 and 2014, but not in 2003. Compared with non-assisted deliveries, children whose births were assisted by a medical doctor had higher chances of being breastfed early in 1998 and 2014 only. In 2014 only, children whose births were assisted by TBAs had a higher likelihood of achieving EIBF compared with non-assisted deliveries. Compared with home-delivered children, being born in a private health facility delayed EIBF in 2008 only. Except for 2003 and 2008, where childbirth order was not statistically significant, the results revealed that being an infant who was not firstborn facilitated EIBF. Maternal age was statistically significantly associated with EIBF in 1998, 2003 and 2014 only. The following factors were statistically significantly related to EIBF in just one survey round: multiple deliveries (1998 only), birth size (1998 only), place of delivery (2008 only), household wealth (2003 only) and place of residence (1998 only).

The gender of the baby, maternal education, maternal exposure to newspaper, radio and TV, and maternal religious affiliation were not statistically significantly associated with EIBF in any of the four surveys.

## Discussion

This study was conducted to develop insights into facilitators and risk factors associated with EIBF in Ghana between 1998 and 2014. We found that the trend in the proportion of children who achieved EIBF in Ghana and the basic forecast analysis based on the observed trend revealed that Ghana is likely to achieve its EIBF target of 85% in 2044. The huge increase in EIBF proportion between 1998 and 2003 can be attributed to the health policy interventions between 2000 and 2004. By the end of 2001, community mutual health organizations started becoming popular, which grew from 47 in 2001 to 168 by 2003.^23^ These developments laid a solid foundation for the design, piloting and implementation of the national health insurance scheme by 2003.^23^ These policy directions allowed many Ghanaians to access healthcare in institutions after paying a one-time premium.^23^ It is believed that this health policy climate resulted in better overall health outcomes for Ghanaians.^23^ Considering the recent estimate of about 55%, it may be said that only about 6 in 10 children born in Ghana benefited from the positive effects of EIBF on child survival, cognitive development and child–mother bonding.^1–3^^,^^8^^,^^9^

These results suggest that delayed initiation of breastfeeding should be regarded as a public health problem in Ghana and given deserved immediate policy attention. There are countries in sub-Saharan Africa and South Asia with enviable records of EIBF proportion estimates from whom Ghana could learn. For instance, 85%, 80.5% and 90.3% of babies born in Burundi, Rwanda and Sri Lanka, respectively, were breastfed early.^7^

We found that delivery by caesarean section was a reliable predictor of EIBF, with the probability of EIBF decreasing in children who were born by caesarean section. This finding could be attributable to the time usually required for the mother to recover from the sedative and semi-conscious effects associated with anaesthesia and the time spent addressing the possible complications that the mother and/or neonate may have suffered from the caesarean section. The result of our study corroborates previous studies that have reported caesarean delivery to be negatively associated with EIBF.^14^^,^^15^^,^^18^^,^^24–26^

We observed from our study that, compared with singletons, children who were the product of multiple births were at risk of delayed initiation of breastfeeding in 1998 only, making it an unreliable predictor of EIBF in Ghana. Although we found the type of birth to be significantly associated with EIBF in 1998 only, studies that assessed this association between 2013 and 2016 did not find it to be statistically significant in a multiple variable regression model.^26^^,^^27^

We further observed that household wealth was not associated with EIBF in any of the survey rounds except 2003, making it an unreliable predictor of EIBF. A similar study in Kenya also concluded that household wealth was an unreliable predictor and not significantly associated with EIBF in any of the DHS rounds used in the study.^14^ Despite the general agreement between our findings and those of the Kenyan study,^14^ there are findings reported from other contexts that are contradictory to this observation.^22^^,^^24^^,^^25^ These studies found an association with EIBF, albeit negative. For example, reports from studies conducted by Khanal et al.^15^ and Senarath et al.^28^ indicate that children from rich households were less likely to achieve EIBF. It is therefore possible that associations between household wealth and EIBF may be contextual. This suggests that further research is needed to generate deeper insights into this specific relationship to aid intervention efforts.

Except for 1998, our analysis showed no significant rural–urban difference in the practice of EIBF over the period under study. This contradicts results reported in previous studies from other contexts.^14^^,^^15^^,^^17^^,^^25^^,^^26^ However, the region of residence was a reliable predictor of EIBF and this concurs with reported findings from elsewhere.^14^^,^^15^^,^^17^^,^^26^^,^^29^ We observed that children who were born in the Upper East Region of Ghana had higher probabilities of being breastfed within the first hour of birth. Considering that the Upper East Region has been a hotbed of health research activity and a piloting base for many health programmes and interventions in Ghana, the population there may have benefited from education on the importance of EIBF. For instance, the region was the pilot area for the Community-based Health Planning and Services programme aimed at achieving universal health coverage and access to essential primary health services.^30^ This pragmatic strategy may have contributed to the improved practice of EIBF in the region. Also, the region is home to the Navrongo Health Research Centre, which, in addition to its quarterly household surveys, regularly embarks on health education programmes in the region.

Children of mothers who were assisted by nurses, midwives, doctors or TBAs (for the most recent survey) were more likely to achieve EIBF. The most probable explanation for this finding could be that midwives, nurses and doctors have knowledge and skills to educate mothers on EIBF as well as supervise its practice. Many TBAs in Ghana have benefited from health training and supervision,^31^^,^^32^ which may have contributed to the positive effect of TBA-assisted deliveries on EIBF in Ghana. This finding is consistent with those that have been reported from studies elsewhere.^17^^,^^25^^,^^33^ The implication is that an increase in access to assistance during deliveries would help increase the practice of EIBF.

The following are some important factors, although not consistent across all the surveys, that were associated with EIBF in the most recent survey: mother’s age and childbirth order. We observed that children of non-teenage mothers were at risk of not achieving EIBF in Ghana. The protective effect of children of teenage mothers achieving EIBF could be that these mothers may be more likely to comply with the expert directive on practising EIBF compared with their older counterparts. Also, the study observed that infants who were not firstborn had a higher likelihood of achieving EIBF. After the first birth, mothers may have accepted and were willing to practice EIBF as communicated through health education during the antenatal period of successive deliveries. These results are broadly in agreement with the results of other studies.^17^^,^^29^^,^^34^

These factors were not statistically significantly associated with EIBF in our study: gender of the baby, maternal education, maternal exposure to mass media and mother’s religion. The statistically significant associations of these factors with EIBF are inconclusive in the literature.^14–17^ For instance, studies have found no significant effect of the gender of the baby,^14–16^ maternal religious affiliation^16^ and maternal education^24^ on children’s achievement of EIBF. In a study conducted by Matanda et al.^14^ using three rounds of DHS data (1998, 2003 and 2008), they found that, except for watching the television, which was not related to EIBF in any of the survey rounds, the mother’s exposure to newspaper and radio were statistically significantly associated with EIBF in 2003 and 1998, respectively. Maternal education was found to be significantly associated with EIBF in studies conducted elsewhere.^14^^,^^16^ Our finding of no association between maternal education and EIBF may be seen as surprising, given the many studies and theories that have suggested that education is a key social determinant of child nutrition and health outcomes.

### Recommendations

The results of our study have public health policy, programme and intervention implications for encouraging mothers to breastfeed their newborns within the first hour of birth. We propose that the Ministry of Health, non-governmental organizations and other development partners in Ghana should pursue policies that aim at intensifying health education among childbearing women. We further propose that policies and programmes that encourage women to deliver in health facilities should be strengthened, while nurses, midwives, TBAs and doctors should continually emphasize the importance of mothers breastfeeding their babies within the first hour of birth. Considering the negative effect of delivery by caesarean section on EIBF, we recommend that health facilities should anticipate this situation and institute measures that promote EIBF immediately after the caesarean section, provided the conditions of the mother and the neonate are stable. The mass media had no impact on EIBF due to the near-absence of EIBF-promoting programmes. Therefore, the government of Ghana, through the Ministry of Health, should embark on such campaigns. Also, mother- and baby-friendly programmes in health facilities with maternal services should be improved to facilitate EIBF.

### Strengths and limitations

The GDHS data are nationally representative and we employed complex sample analyses that accounted for multistage sampling design and weighting, which makes our estimates accurate and generalizable for the entire country. Another important strength is that we have unravelled the factors that consistently predict EIBF, giving policymakers a sense of direction about the most important risk factors to address and the protective factors to improve. One potential limitation is the possibility of recall bias on the part of mothers in answering the survey question on EIBF, which we have no control over in our analyses.

## Conclusions

In conclusion, the study revealed that the proportion of children achieving EIBF in Ghana was consistently lower than the national and global targets. Delivery by caesarean section and the region of residence were consistently associated with EIBF across the four surveys. Other salient findings were that being an infant who was not firstborn, as well as deliveries assisted by healthcare professionals and TBAs, facilitated EIBF in the most recent survey. To achieve Ghana’s national target, there is a need for pragmatic efforts at various levels of the healthcare system to vigorously promote the practice of EIBF.
