# Corrigendum to: Consistency of the determinants of early initiation of breastfeeding in Ghana: insights from four Demographic and Health Survey datasets

**DOI:** 10.1093/inthealth/ihaa025

**Published:** 2020-05-04

**Authors:** Precious A Duodu, Henry O Duah, Veronica M Dzomeku, Adwoa B Boamah Mensah, Josephine Aboagye Mensah, Ernest Darkwah, Pascal Agbadi

**Affiliations:** Department of Nursing, Faculty of Allied Health Sciences, College of Health Sciences, Kwame Nkrumah University of Science and Technology, Private Mail Bag, Kumasi, Ghana; Research Department, Foundation of Orthopaedic and Complex Spine Hospital, Post Office Box KD 779 Kanda, Accra, Ghana; Department of Nursing, Faculty of Allied Health Sciences, College of Health Sciences, Kwame Nkrumah University of Science and Technology, Private Mail Bag, Kumasi, Ghana; Department of Nursing, Faculty of Allied Health Sciences, College of Health Sciences, Kwame Nkrumah University of Science and Technology, Private Mail Bag, Kumasi, Ghana; Child Health Directorate, Komfo Anokye Teaching Hospital, P.O.Box 1934 Adum-Kumasi, Ghana; Department of Psychology, University of Ghana, Post Office Box LG 84, Legon, Accra, Ghana; Department of Nursing, Faculty of Allied Health Sciences, College of Health Sciences, Kwame Nkrumah University of Science and Technology, Private Mail Bag, Kumasi, Ghana

In Table 1 of the above article, the values for `Singleton' and `Multiple birth' in 2003 were inverted. This has now been corrected as follows:

2003

**Type of Birth**

Singleton 3494 (96.0)

Multiple birth 145 (4.0)

